# Actin Cytoskeleton Role in the Maintenance of Neuronal Morphology and Long-Term Memory

**DOI:** 10.3390/cells10071795

**Published:** 2021-07-15

**Authors:** Raphael Lamprecht

**Affiliations:** Sagol Department of Neurobiology, Faculty of Natural Sciences, University of Haifa, Haifa 3498838, Israel; rlamp@research.haifa.ac.il

**Keywords:** learning and memory, dendritic spines, actin cytoskeleton

## Abstract

Evidence indicates that long-term memory formation creates long-lasting changes in neuronal morphology within a specific neuronal network that forms the memory trace. Dendritic spines, which include most of the excitatory synapses in excitatory neurons, are formed or eliminated by learning. These changes may be long-lasting and correlate with memory strength. Moreover, learning-induced changes in the morphology of existing spines can also contribute to the formation of the neuronal network that underlies memory. Altering spines morphology after memory consolidation can erase memory. These observations strongly suggest that learning-induced spines modifications can constitute the changes in synaptic connectivity within the neuronal network that form memory and that stabilization of this network maintains long-term memory. The formation and elimination of spines and other finer morphological changes in spines are mediated by the actin cytoskeleton. The actin cytoskeleton forms networks within the spine that support its structure. Therefore, it is believed that the actin cytoskeleton mediates spine morphogenesis induced by learning. Any long-lasting changes in the spine morphology induced by learning require the preservation of the spine actin cytoskeleton network to support and stabilize the spine new structure. However, the actin cytoskeleton is highly dynamic, and the turnover of actin and its regulatory proteins that determine and support the actin cytoskeleton network structure is relatively fast. Molecular models, suggested here, describe ways to overcome the dynamic nature of the actin cytoskeleton and the fast protein turnover and to support an enduring actin cytoskeleton network within the spines, spines stability and long-term memory. These models are based on long-lasting changes in actin regulatory proteins concentrations within the spine or the formation of a long-lasting scaffold and the ability for its recurring rebuilding within the spine. The persistence of the actin cytoskeleton network within the spine is suggested to support long-lasting spine structure and the maintenance of long-term memory.

## 1. Introduction

Memory can last for years. The prevailing hypothesis is that memory is encoded by a neuronal circuit, and therefore, this circuit needs to be preserved to maintain the memory. This circuit includes, in many instances, excitatory and inhibitory synapses in excitatory neurons. Most of the synaptic excitatory transmission, but also some of the inhibitory synaptic transmission, occurs in dendritic spines that are small extensions of the dendrite. It was found that the morphological structure of the spine can affect the transmission of the synaptic signal into the neuron and can determine its activity. Moreover, the number of dendritic spines and their location in the neuron contribute to the way the inputs that arrive at the neuron activate it. Thus, dendritic spines can sculpt and determine the function of the neuronal circuit that encodes memory. It also implies that if the neuronal circuit that encodes a lasting memory is preserved, then the overall influence of the dendritic spines on the activity of the neurons, and hence their morphology and distribution within the circuit, needs to be maintained. Indeed, studies have shown that dendritic spines formed after learning can be preserved for a very long time, exhibiting a strong correlation between the number of the maintained new spines and the strength of memory. Long-lasting elimination of spines can also play a role in shaping the neuronal circuit that forms the memory trace. These observations suggest that long-term memory maintenance is subserved by long-lasting dendritic spines. Dendritic spines morphology is mediated by the actin cytoskeleton structure. However, the actin cytoskeleton is very dynamic, and the lifetime of proteins that regulate and preserve the actin cytoskeleton structure is relatively short. How, therefore, is the structure of dendritic spines preserved by the actin cytoskeleton to maintain long-term memory? This review discusses this topic and suggests models that can resolve the issue. 

### 1.1. The Functions of Dendritic Spines in Neurons

Dendritic spines are protrusions extending from the neuronal dendrites. Most of the excitatory transmission in excitatory neurons is located in spines [1,2]. Some of the inhibitory transmission can also be found in spines [3]. The spine contains the spine neck and head at different sizes and proportions. The head contains multiple proteins that receive the synaptic transmission, interacts with the presynapse, allows the influx of ions through the membrane and transmits synaptic information to the cytoplasm. The spines can be found in different shapes and can be classified morphologically as stubby spines lacking a neck, thin spines that contain a long neck with an apparent head or mushroom spines with a big head and thick neck [4].

It is believed that spine number and density, as well as distribution along the dendrite and their morphology, contribute to their influence on signal transmission, propagation of the stimulus and neuronal activity. The size and geometry of the spine head and neck contribute to synaptic signal compartmentalization, strength and specificity. Spine head: The volume of a spine head is proportional to the area of the spine’s postsynaptic density (PSD), to the presynaptic partner and the number of synaptic AMPA receptors (AMPARs) and the amplitude of the AMPAR-mediated current [5,6,7]. Therefore, the morphology of the spine head is tightly coupled with synaptic transmission. Spine Neck: It has been suggested that spines serve as electrical compartments because of the resistance at the spine neck. The electrical compartmentalization leads to amplification of excitatory postsynaptic potentials (EPSPs) locally within the spine, a voltage gradient between the spine and the dendritic shaft and a reduction in dendritic and somatic EPSPs compared with those in the spine. The shape of the spine neck determines the fate of Ca^2+^ that enters the spine heads through NMDARs. Larger spines have necks that permit a greater efflux of Ca^2+^ into the dendritic shaft, whereas smaller spines display a larger increase in [Ca^2+^]_i_ within the spine compartment because of a smaller Ca^2+^ flux through the neck [8]. This allows small spines to be the preferential sites for isolated induction of long-term potentiation. The spine neck may affect the propagation of the stimulus along the neuron. For example, it has been shown that stimulation of spines with longer necks produces smaller EPSPs at the soma [9,10]. On the other hand, spines experiencing a rapid shrinkage in spine neck length correlate with an increase in the somatically recorded uncaging potential [10]. Spines activity integration and distribution in neurons: Spines may be involved in the way synaptic inputs to the neuron integrate to induce neuronal activation. For example, experiments activating 2–3 [11]), 7–10 [12] or up to ∼20 spines [13] have shown that glutamate excitatory inputs into spines integrate linearly before additional inputs generate a dendritic spike, whereas inputs delivered into the dendritic shaft in the same compartments integrate sublinearly. These results indicate that spines function as electrical isolators to prevent input interaction, and thus, they produce linear arithmetic of excitatory inputs. Linear integration could be essential for neuronal information processing [14].

### 1.2. Dendritic Spines and Memory Formation and Maintenance

Spines may be involved in long-term memory formation and its storage. If spines number, distribution and morphology encode the memory trace, then spines need to be stable for a long period to maintain the long-term memory. Indeed, it has been shown that spines can be stable for a long period. In the adult mouse, visual cortex spines remain stable for months [15]. However, many spines underwent marked changes in length or head diameter over time. It has been shown, in the auditory cortex, that spines last for months but may change in size and that the magnitude of changes in spine size is proportional to the size of the spine. Thus, changes in spine sizes are multiplicative, an observation that provides insights into the emergence of the log-normal distribution of spine sizes in the neocortex [16].

Learning can facilitate the formation and elimination of spines in adults, and these changes in morphology may last for a long period. For example, fear conditioning causes dendritic spine elimination in the frontal association cortex (FrA) [17]. These changes are enduring and last for days (spines were examined nine days after conditioning). In addition, the authors showed that fear conditioning memory extinction training leads to spine formation in the FrA. These changes are also long-lasting (the changes were tested seven days after extinction training). Spine elimination and formation after fear conditioning and extinction occur on the same dendritic branches in a cue- and location-specific manner. In another study, it was shown that motor learning (rotarod training) and novel sensory experience promote rapid dendritic spine formation [18]. A small fraction of new spines induced by novel experience was preserved for months. Moreover, the animal’s performance at day seven strongly correlated with the percentage of new spines that were formed during the first two-day training and that lasted for seven days, showing a strong correlation between the maintained new spines and learning and suggesting that these spines are important for the persistence of the new cortical circuits that underlie the lasting acquired motor skills. In addition, it has been shown that the new spines induced by forelimb reaching task learning are preferentially stabilized during subsequent training and endure long after training stops [19]. Memory can also affect fine spines morphology; for example, fear conditioning decreases spine head volume in the lateral amygdala (LA) and leads to an increase in the PSD area in the smooth endoplasmic reticulum (sER)-free spines [20]. In another study, investigators created an AS-PaRac1 probe that specifically labelled the enlarged and newly generated spines (‘structurally potentiated spine’) [21]. Mice trained for motor tasks exhibited significantly more structural potentiation spines compared with the non-trained mice. Structural potentiation spines labeled during the learning period were more likely to be preserved (measured for two days) than those labeled when the animals were not subjected to training. Photostimulation of the AS-PaRac1 (which leads to activation of Rac1 GTPase, which may regulate actin, located within the AS-PaRac1 construct) leads to the shrinkage of the AS-PaRac1-containing spines. If this AS-PaRac1 photoactivation protocol is applied a day after training to the motor cortex, it erases the acquired motor task skills. Thus, the newly acquired motor skill depends on the formation of the newly structurally potentiated spines.

In addition, the actin cytoskeleton and its regulatory proteins are involved in brain diseases that lead to spines morphological dysfunctions and memory impairments, such as Alzheimer’s Disease [22,23,24,25,26]. 

## 2. Actin Cytoskeleton Mediates Spine Formation, Spine Elimination and the Morphology of Existing Spines

### 2.1. Actin Supports Spines Morphology

The aforementioned observations show that spines are formed or eliminated after learning and that learning can also induce subtler morphological changes in spines. What are the molecular components that support spine subtle alteration and induce spine formation or elimination? The actin cytoskeleton and their regulatory proteins control spine morphology. Dendritic spines are enriched with filamentous actin (F-actin), and the shape of the spine is subserved by the actin cytoskeleton [27]. The spine actin cytoskeleton contains a mixture of linear and branched actin networks, which extend from the base of the spine through the neck and head to the spine’s top, which includes the postsynaptic density (PSD). This actin cytoskeleton opposes the spine membrane and, therefore, mediates and sculpts the spine shape. Periodic F-actin structures shape is found in the neck of dendritic spines [28]. These periodic actin lattices are found in the neck of nearly all dendritic spines, including the mushroom-like spines with a long thin neck and less mature filopodia-like spines. The actin rings may provide mechanical support to the spine neck. Thus, to maintain the neck structure, important for the spine features and conduction of signals, the spine’s actin ring structures may need to be preserved. Changes in the actin cytoskeleton affect spine morphology. For example, glutamate uncaging induces enlargement of stimulated spines, which is dependent on actin polymerization [29]. Long-term potentiation (LTP) induction shifts the G-actin/F-actin ratio toward F-actin in dendritic spines of rat hippocampal neurons, which leads to an enlargement of spine volume. In contrast, long-term depression (LTD) induction shifts the ratio toward G-actin in dendritic spines and results in spine shrinkage [30]. The actin cytoskeleton dynamic and structure is regulated in spines by signaling molecules (e.g. [31,32,33,34,35,36]). In addition, it has been shown that changes in actin structure and dynamics can be affected by synaptic activation through actin regulatory proteins, forming the synapse–actin regulatory proteins–actin cytoskeleton–spine morphology connection. Synaptic receptors, such as glutamate receptors, Eph receptors and adhesion molecules (e.g., cadherin, L1-CAM), and signaling molecules that participate in spine morphogenesis and memory formation are functionally linked with these actin regulatory proteins (e.g., [37,38]). 

### 2.2. Actin Is Involved in the Stabilization of Spines

Newly formed spines may be stabilized or eliminated with time, leaving a stable fraction of enduring spines. It has been shown that tasks that lead to long-term memory induce the formation of new spines. Most of the spines are eliminated with time, but some spines are preserved for months. The fraction of spines preserved is correlated with training intensity, suggesting that these spines are involved in forming an enduring memory trace [18]. Another study showed that in control mice, newly formed spines were eliminated with time, whereas in motor task-trained mice, more newly formed spines were preserved [19]. These results indicate that the maintaining of memory may involve the stabilization of new spines and preventing them from elimination.

Why are specific spines preserved while others are eliminated? What causes the elimination of spines? One possibility is that spines may be formed initially, but the connectivity with the pre-synapse is not strong enough and does not lead to active actions in the spines that will prevent their elimination. Thus, ongoing activity at the spine is needed to preserve it through maintaining the actin network. For example, activation of spines by presynaptic stimulation leads to their stabilization, while neighboring spines that are not activated shrink, and some are eliminated [39]. This activity-induced spine fate differentiation requires cadherin/catenin-dependent adhesion. Moreover, enhancing cadherin/catenin adhesion on a spine in vivo is sufficient to make it more mature and to destabilize and/or eliminate neighboring spines. Actin reorganization is needed to coordinate spine fate differentiation and β-catenin redistribution.

Activation of glutamate receptors can also affect actin structure, stabilizing the spine structure and preventing it from retraction. A study has shown that spine density and length in CA1 pyramidal cells are reduced after Schaffer collaterals transection or after the application of AMPA receptor antagonists or a botulinum toxin to an unlesioned culture. Loss of spines induced by a botulinum toxin or lesion was prevented by the application of AMPA [40]. The authors concluded that spontaneous AMPA receptor activation by vesicular glutamate release is sufficient to maintain dendritic spines. Actin cytoskeleton dynamics in spines are potently inhibited by the activation of glutamate receptors. Activation of AMPA and NMDA receptors inhibited actin-based protrusive activity from the spine head so that spine morphology became both more stable and more regular [41].

### 2.3. Regulation of Actin Is Involved in Controlling the Retraction of Spines

As described above a possible way of affecting neuronal activity and memory trace is by eliminating spines. Spine elimination was observed in the motor cortex when mice were subjected to motor task training [18]. The elimination of spines over seven days strongly correlated with the animal’s performance on day seven. This finding and the fact that the eliminated spines probably had connections with axons suggest that learning leads to active pruning of spines. Another study shows that fear conditioning leads to spine elimination after 48 h in the frontal association cortex (FrA) [17]. Spine elimination is long-lasting and can be detected when measured after nine days. The percentage of spine elimination correlated with the degree of freezing responses to the tone CS. Thus, fear conditioning causes rapid and long-lasting spine elimination in FrA.

Spine elimination may result from changes in the actin network and involve alterations in actin regulatory proteins concentrations and activities in the spine. For example, synaptopodin (SP) stabilizes F-actin, and spines containing SP survive longer than spines without SP. It was further shown that mature spines that underwent pruning first lost SP before disappearing [42]. Thus, these observations suggest that removal of SP is a necessary step before the actin cytoskeleton of a spine can be disassembled and the spine can be pruned.

It can be speculated that the major difference between formation and elimination of spines is that spine elimination requires no stabilization to maintain their disappearance from the dendrite, whereas spines preservation needs molecular activities for long-lasting maintenance.

## 3. The Role of Actin in Maintaining Spines and Long-Term Memory

Evidence is available showing that actin polymerization is needed for the maintenance of spines and long-term memory. For example, infusion of Latrunculin A (LatA prevents the incorporation of G-actin into dynamic F-actin, [43]) into the basolateral amygdala complex (BLC) two days after conditioned place preference (CPP) training [44] impaired CPP long-term memory. The study further showed that spines density in BLC increased by CPP training and that LatA infusion into BLC two days following training reduced spines density in CPP-paired animals, but not in control animals. Thus, the study implies that memory maintenance is supported by constitutive cycling of actin filaments that maintain spine stability.

Nonmuscle myosin II is also required for the maintenance of CCP memory [44]. Microinjection of intra-BLC Blebbistatin (Blebb), an inhibitor of nonmuscle myosin II motor activity [45], before a retrieval test led to an immediate and persistent disruption of CPP memory.

## 4. How Can a Dynamic Actin Cytoskeleton Support the Maintenance of the Changes in Dendritic Spines Induced by Learning?

**Figure 1:**
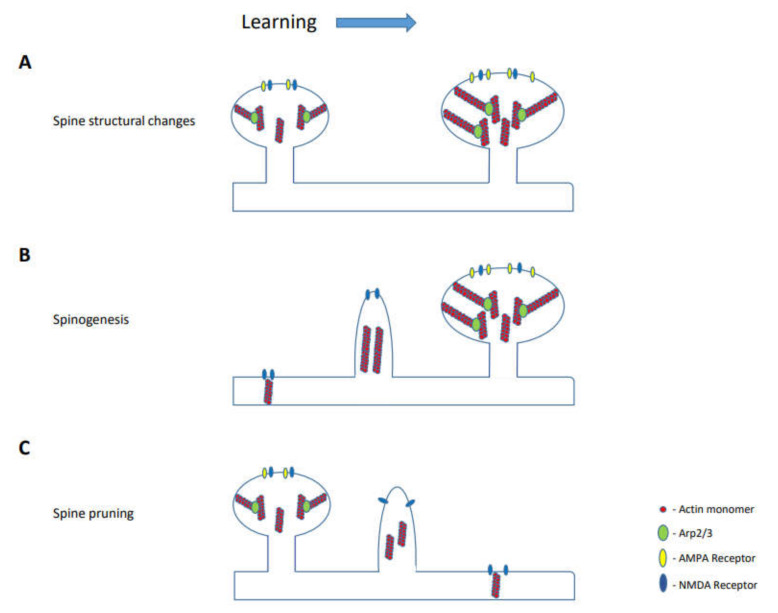
Learning leads to morphogenesis of existing spines, formation of new spines and elimination of spines. (**A**). The formation of a new actin network during learning in existing spines leads to a change in their morphology. (**B**). Stimulation of dendritic receptors during learning promotes F-actin accumulation at nascent synaptic sites, leading to the outgrowth of filopodia and mature spines (e.g., [46,47]. (**C**). Spine elimination may result from destabilizing the actin network, which can involve alterations in the stabilization or the recurrent formation of the F-actin network by actin regulatory proteins.

It has been shown above that learning leads to spines formation and elimination and that these changes last for a long-time. Moreover, the magnitude of the changes and their persistence correlate with memory strength. In addition, learning leads to changes in the morphology of preexisting spines (Figure 1). These observations strongly suggest that spines morphogenesis and their persistence are involved in memory formation and storage, respectively. Actin cytoskeleton supports the morphology of spines. It therefore may support memory maintenance through stabilizing the alterations in spines that were induced by learning, by: (1) preserving the alterations of the existing spine’s fine structure (e.g., head and neck morphology), (2) maintaining specific newly formed spines and (3) retracting specific spines. These functions are hypothesized to be involved in the stabilization of the neuronal network that underlies the long-term memory trace. These observations beg the question: how can the dynamic actin cytoskeleton and the relatively rapid turnover of actin-regulatory proteins support the long-lasting morphology of spines and the neural network?

### 4.1. Maintaining Spine Structure in Light of the Rapid Actin Dynamics and Relatively Short Half-Life of Proteins

The aforementioned observations indicate that some spines may be dynamic, appearing and retracting at a high rate, and some spines may have rapid internal fluctuations in structure over time. However, a fraction of spines induced by learning persist for many days and months. Moreover, manipulation of the long-lasting spines morphology can affect long-term memory. These results strongly suggest that spines formation and their particular morphology are involved in encoding the long-term memory trace. Thus, it is inferred that if the memory trace lasts for many days, so too must the spines involved in encoding memory persist for that enduring period. The actin cytoskeleton can support the maintenance of spine structure. However, there are several obstacles associated with the rapid kinetics of the actin cytoskeleton and its regulatory proteins that need to be overcome so that actin can stabilize the structure of a spine. First, the actin cytoskeleton is very dynamic. F-actin at the spine tip and periphery are dynamic, cycling between G-actin and F-actin with a turnover of tens of seconds. Actin in the center and base of the spine is less dynamic and more stable, exhibiting a turnover in the range of tens of minutes [48]. Over 80% of F-actin in spines turns over every minute [49]. How can the actin cytoskeleton support the spine structure if it so dynamic? The second issue is protein turnover. In the brain proteins’ half-life ranges from few hours to few days [50], and although the half-life of neuronal proteins can last for many days, the turnover of synaptic proteins is in the range of few days [51]. How can spines and their actin network retain their structure if the turnover of proteins, which are involved in regulating and maintaining these structures, is relatively fast?

**Figure 2:**
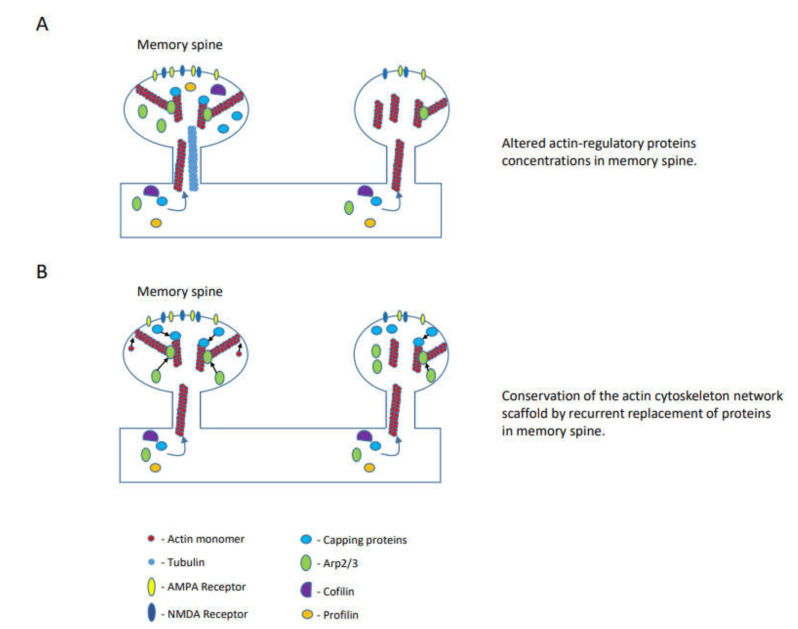
Actin networks in spines can be maintained after learning by preserving actin-regulatory proteins concentrations in a spine’s nano-domain to keep the general network intact, by rebuilding the scaffold actin network created by learning or by a combination of both. (**A**). Actin-regulatory proteins concentrations are altered in the memory spine and determine the general structure of the actin network. (**B**). The actin network is altered by learning in memory spines and serves as a scaffold for recurrent rebuilding and conservation of the network.

Therefore, the neuron should overcome the fast dynamic of the actin filaments and network and the fast protein turnover to form a long-lasting actin cytoskeleton network that can support the enduring spine structure. Thus, after learning and during consolidation, a molecular process should be engaged to preserve the new actin cytoskeleton structure, formed by learning, within the spine so that spine structure can be consolidated and maintained. Several mechanisms (Figure 2) can be considered: (1) maintaining the relative relationships between the branched and linear actin filaments in each of the spine nano-domains so that the structure of the overall actin network will be preserved. This mechanism is not dependent on the existing network but on the concentrations and activities of the actin-regulatory proteins within the spines that keep the general actin network structure consistent. These concentrations and activities of the actin regulatory proteins are altered by learning and preserved during maintenance. (2) The actin cytoskeletal network, created by learning, serves as a scaffold. The network structure is kept by adding new proteins to replace proteins destined for degradation. Both mechanisms depend on the supply of new proteins, but the first one depends on keeping the concentrations and activities of the proteins in the spines in equilibrium and, thus, requires a mechanism that supplies the proteins specific to the memory spines. The second mechanism does not depend on the supplies of the proteins specific to the memory spines, and it depends solely on the initial structure of the network that was created by learning. Is there evidence that supports these models?

### 4.2. Translocation of Proteins into Activated Spines to Initiate Spine Morphogenesis and the Subsequent Maintenance of the Morphological Change

As suggested above, spine morphology can be initially altered (by learning) and preserved (during memory maintenance) by changing the actin cytoskeleton network and maintaining these changes in the spine, respectively. This can be done by altering the concentrations of actin regulatory proteins in the spine and keeping these concentrations over time. It has been shown that synaptic activation leads to the insertion of actin-regulatory proteins into spines (Table 1). For example, Bosch et al., 2014 [52] have shown that induction of an LTP in single dendritic spines by glutamate uncaging led to a persistent enlargement of the spine and increase of synaptic transmission. They also found that L-LTP leads to the translocation of actin-regulatory proteins into the spine and to the change in their relative composition in spines. These proteins are known to modify F-actin through severing (cofilin), branching (Arp2/3) or capping (Aip1). Thus, the composition of the actin-regulatory proteins in the spine is altered after synaptic stimulation that leads to changes in morphology and transmission. The new protein composition may lead to alterations in the actin network structure by changing and creating linear and branched actin filaments. Such changes in network properties can affect spine structure, e.g., a more branched actin network can lead to a spine with a larger head. An example of how changing the concentration of actin regulatory proteins can lead to alterations of the actin network comes from the observation that increasing the concentration of the capping proteins (CPs) leads to an increase in the Arp2/3-mediated branching of the actin cytoskeleton network [53]. Nucleation promoting factor (NPF)-bound actin monomers can associate with barbed ends, leading to filament elongation, or they can participate in nucleation. Elongation is kinetically favored over nucleation. Capping proteins can promote nucleation by eliminating the competition from barbed ends. Thus, adding capping proteins may facilitate the formation and maintenance of a more branched actin network. On the other hand, factors like VASP (vasodilator-stimulated phosphoprotein), which has been shown to antagonize CP, decrease the density of Arp2/3-dependent branches in actin comet tails [54]. This can promote the transformation from dendritic/lamellipodial to bundled/filopodial actin architecture in vivo [55].

In addition to a regulation of the actin cytoskeleton by binding directly to the actin cytoskeleton, the regulatory proteins may also be regulated by post-translational modification, such as phosphorylation. For example, the activity of cofilin is regulated by phosphorylation, where LIM kinase-dependent phosphorylation suppresses the activity of cofilin, while cofilin dephosphorylation by slingshot phosphatase leads to cofilin activation [56,57,58,59]. The WAVE Regulatory Complex (WRC) that activates the Arp2/3 actin nucleator is also regulated by phosphorylation [60]. Such a posttranslational modification of actin regulatory proteins affects spines morphology (e.g., WAVE1 [61]; Limk1 [62]). Thus, the composition of other regulatory proteins that affect actin-regulatory proteins by post-translational modification in the spines may affect spines stability, in particular in response to external stimulation.

The translocation of actin regulatory proteins into spines has also been induced by learning. Fear conditioning leads to the movement of profilin into dendritic spines in the lateral amygdala (LA). These profilin-containing spines undergo enlargements in their postsynaptic densities (PSDs) [63]. Profilin is also shown to be responsive to other neuronal stimuli. For example, profilin is targeted to the spine head when postsynaptic NMDA receptors are activated [64]. Profilin (YFP-PFN2a) is enriched in spines of hippocampal neurons upon stimulation [65]. Profilin I is localized at synaptic sites in an activity-regulated manner [66]. VASP binding to profilin is needed for profilin-mediated stabilization of the actin cytoskeleton and dendritic spine morphology [64]. Microinjection of poly-proline peptide [G(GP_5_)_3_] into LA, to interfere with VASP binding to profilin, impaired long-term, but not short-term, fear memory formation [67].

**Table 1:** Examples of actin-regulatory proteins that are translocated into dendritic spines and are involved in spine morphogenesis or stabilization.

Actin-Regulatory Protein	Stimulation/System	Effect	Reference
Profilin	Postsynaptic NMDAreceptors and LTP and LTD/Cultured hippocampalneurons.	Actin-based changes in spine shape are blocked, and the synaptic structure is stabilized.	[64]
Profilin	Fear conditioning/Lateral amygdala.	Spines undergo enlargements in their postsynaptic densities (PSDs).	[63]
Cofilin	Initial phase after LTP in a single dendritic spine with two-photon (2P) uncaging of glutamate/Rat hippocampal organotypic slice culture.	The spine undergoes enlargement.	[52]
Arp2/3	Initial phase after LTP in a single dendritic spine with two-photon (2P) uncaging of glutamate/Rat hippocampal organotypic slice culture.	The spine undergoes enlargement.	[52]
Aip1	Initial phase after LTP in a single dendritic spine with two-photon (2P) uncaging of glutamate/Rat hippocampal organotypic slice culture.	The spine undergoes enlargement.	[52]

### 4.3. Trafficking of Proteins into Spines

The preservation of the composition of actin regulatory proteins in spines requires the persistent trafficking of specific proteins but not others into specific “memory spines”. Proteins can be translocated into spines using Myosin motor proteins that use actin filaments [68]. For example, the Flr protein, which functions as a dominant-negative MyoVa, sequesters cargo (such as PSD-95, PSD-93 and SAP102) and blocks its transport to the PSD [69]. Flr leads to an increase in the number of filopodia and to a decrease in mushroom spines in apical dendrites of hippocampal neurons. The formation of a new actin cytoskeleton in these spines after activation and its preservation may lead to the formation of novel routes and their maintenance for translocating the actin regulatory proteins into the spines by proteins, such as Myosin, that use these new routes. This can serve as a perpetual mechanism for the constant delivery of actin regulatory proteins into spines and within the spine. These routes can also deliver specific mRNA for local translation of proteins in spines [68]. Another possibility of directing the proteins into specific spines is via microtubules. It has been shown that activity-dependent actin remodeling at the base of spines promotes microtubule entry [70]. Microtubules entering dendritic spines provide a direct route for motor-driven transport of specific synaptic cargo into spines (e.g., [71,72]). Although microtubule entry into the spine can be transient, a stable actin structure in the base of the spine may promote a stable microtubule or its reentry. Keeping stable actin in the spine’s base can promote the entrance of the microtubule or form actin routes for myosin entry into the spines or both to internalize selective proteins that will cause the stabilization of these structures in the spine’s base and other parts. This positive reinforcement and recurrent activities could support the lasting proteins concentrations in spines, actin cytoskeleton network structure stability, spines stability and long-term memory.

## 5. Conclusions and Future Directions

The prevailing hypothesis is that the persistence of long-term memory requires a stable underlying neuronal network. Dendritic spines contain most of the excitatory synapses in excitatory neurons, and their morphology affects synaptic transmission. Therefore, it is suggested that stable dendritic spines morphology may underlie the maintenance of the neuronal network and long-term memory. The morphology of dendritic spines is supported by the actin cytoskeleton. However, the actin cytoskeleton is dynamic, and the turnover of proteins that control this actin cytoskeleton network is relatively fast. To overcome this and to keep the actin cytoskeleton structure within the spines in a relatively stable form, for the maintenance of the general structure of the spine, two models have been suggested: (1) The concentrations of the actin cytoskeleton regulatory proteins within the spine’s nano-domains should be kept consistent. This could be done by funneling specific proteins or mRNAs into the spine by routes created by learning. (2) A structure has been formed during learning and is maintained the same by replacing the actin-binding proteins and actin, which builds the structure and are destined to degradation, with new proteins.

Although there is evidence that supports the first model, i.e., insertion of specific proteins into activated spines to control their morphology and the formation of routes of transportation of proteins and mRNAs into the spines, future studies will need to investigate this model. These studies can inspect: (1) the concentrations of proteins in a spine’s nano-domain after activation over time, (2) how altering these concentrations affects a spine’s nano-domain and the spine structure and (3) how the trafficking routes arrive at these nano-domains to deliver the proteins and how these routes remain stable over time. The second model, namely, the formation, by learning, of an actin cytoskeleton network and the constant replacement of proteins to keep the network stable for memory maintenance, should also be studied.
